# The single nucleotide polymorphism rs1814521 in long non-coding RNA *ADGRG3* associates with the susceptibility to silicosis: a multi-stage study

**DOI:** 10.1265/ehpm.21-00338

**Published:** 2022-02-19

**Authors:** Wei Wang, Xiaofeng Chen, Chunping Li, Rui Zhao, Jinlong Zhang, Hong Qin, Miaomiao Wang, Yao Su, Minzhu Tang, Lei Han, Na Sun

**Affiliations:** 1 Department of Occupational Health, Center for Disease Control and Prevention of Wuxi, Wuxi, Jiangsu, China; 2 Department of Quality Management, Center for Disease Control and Prevention of Wuxi, Wuxi, Jiangsu, China; 3 Department of respiratory medicine, Wuxi Eighth People's Hospital, Wuxi, Jiangsu, China; 4 Institute of Occupational Disease Prevention, Jiangsu Provincial Center for Disease Prevention and Control, Nanjing, Jiangsu, China

**Keywords:** Silicosis, lncRNA, *ADGRG3*, rs1814521, Biomarker

## Abstract

**Background:**

This study aimed to evaluate the correlation between long non-coding RNA (lncRNA)-related single nucleotide polymorphisms (SNPs) and susceptibility to silicosis.

**Methods:**

First, RNA-sequencing (RNA-seq) data were comprehensively analyzed in the peripheral blood lymphocytes of eight participants (four silicosis cases and four healthy controls) exposed to silica dust to identify differentially expressed lncRNAs (DE-lncRNAs). The functional SNPs in the identified DE-lncRNAs were then identified using several databases. Finally, the association between functional SNPs and susceptibility to silicosis was evaluated by a two-stage case-control study. The SNPs of 155 silicosis cases and 141 healthy silica-exposed controls were screened by genome-wide association study (GWAS), and the candidate SNPs of 194 silicosis cases and 235 healthy silica-exposed controls were validated by genotyping using the improved Mutiligase Detection Reaction (iMLDR) system.

**Results:**

A total of 76 DE-lncRNAs were identified by RNA-seq data analysis (cut-offs: fold change > 2 or fold change < 0.5, *P* < 0.05), while 127 functional SNPs among those 76 DE-lncRNAs were identified through multiple public databases. Furthermore, five SNPs were found to be significantly correlated with the risk of silicosis by GWAS screening (*P* < 0.05), while the results of GWAS and iMLDR validation indicated that the variant A allele of rs1814521 was associated with a reduced risk of silicosis (OR = 0.76, 95% CI = 0.62–0.94, *P* = 0.011).

**Conclusion:**

The presence of the SNP rs1814521 in the lncRNA *ADGRG3* is associated with susceptibility to silicosis. Moreover, *ADGRG3* was found to be lowly expressed in silicosis cases. The underlying biological mechanisms by which lncRNA *ADGRG3* and rs1814521 regulate the development of silicosis need further study.

**Supplementary information:**

The online version contains supplementary material available at https://doi.org/10.1265/ehpm.21-00338.

## Introduction

Silicosis is an occupational disease of the respiratory system that is usually caused by the long-term inhalation of respirable crystalline silica dust [1]. From 1990 to 2017, the global incidence of silicosis increased by 58.3%, mainly in low- and middle-income countries [2]. Meanwhile, in the United States and England, the age of silicosis onset has declined considerably, thus indicating that individuals in developed countries are also prone to silicosis [3, 4]. Therefore, it is obvious that silicosis has evolved into a global public health issue.

Silicosis is a progressive and incurable disease characterized by pulmonary fibrosis. However, silicosis can be prevented by limiting exposure to dust in the air [5]. Generally, occupational exposure to silica has been identified as the main contributor to silicosis [6], and silicosis progression is strongly associated with the exposure time and silica concentration [7]. In addition, individual genetic susceptibility was also reported to play a significant role in the occurrence and development of silicosis. In particular, single nucleotide polymorphisms (SNPs) have been linked to the occurrence of silicosis [8].

Currently, genome-wide association study (GWAS) is widely used to test the association between SNPs and complex diseases or traits [9]. In a previous three-stage case-control study, the SNP rs73329476 on chromosome 12q15 was identified to have a significant correlation with susceptibility to silica-related pneumoconiosis via GWAS [10]. Moreover, Allen et al. performed GWAS and found that the minor allele A of rs62025270 is associated with increased susceptibility to idiopathic pulmonary fibrosis (IPF) in European populations [11]. However, most of the SNPs identified by GWAS are located in the “desert” or intronic regions and hence may lack biological functions. Both the above-mentioned fibrosis-related SNPs (rs73329476 and rs62025270) are also located in the “desert” region of the genome. Furthermore, GWAS was more frequently seen as a type of association study rather than a causation study and was also regarded as the study of narrowing the scope of the causal gene. Therefore, investigating the potential function of regulatory elements in GWAS-identified region may facilitate the understanding of biological mechanisms of complex diseases.

In recent years, the rapid development of high-throughput sequencing technology has greatly promoted genetic research in RNA biology [12, 13]. According to human genome sequencing analysis, less than 2% of the human genome encodes proteins, and more than 98% of total transcripts are non-coding RNAs (ncRNAs) [14]. Nevertheless, a large number of studies have demonstrated the important functions of ncRNAs [15, 16]. For example, microRNAs (miRNAs) can mediate post-transcriptional gene silencing by binding to target mRNAs [17]. Circular RNAs (circRNAs) can compete with mRNAs and interact with proteins to regulate a variety of biological processes [18]. These findings indicate different types of ncRNAs, which are present throughout the human transcriptome, can regulate gene expression through various mechanisms [19]. Long non-coding RNAs (lncRNAs), which comprise the largest class of ncRNAs in the human transcriptome, span a length of more than 200 nucleotides [20]. Although the functions of lncRNAs have not yet been fully elucidated, their roles in the regulation of biological processes have been widely acknowledged [21]. Several studies have suggested that lncRNA serves as a potential therapeutic target and potential biomarker in various human diseases [22, 23], especially in fibrotic diseases [24]. Sai et al. showed that silica induced changes in the expression of numerous lncRNAs and that these altered lncRNAs affected the occurrence and development of pulmonary fibrosis by regulating the expression of genes in rat lungs [25]. Meanwhile, silencing of the lncRNA *AK131029*, which is overexpressed in patients with silicosis, could inhibit the proliferation and migration of human lung fibroblasts, thereby preventing the progression of pulmonary fibrosis [26]. Additionally, some lncRNAs, including lncRNAs *MALAT1*, *ATB*, and *CHRF*, are involved in a competitive endogenous RNA (ceRNA) regulatory network, which affects the progression of pulmonary fibrosis [27–29].

Both SNPs and lncRNAs play important roles in the development and progression of many diseases, and some studies have reported the influence of SNPs in lncRNAs on many diseases [30, 31]. Li et al. found that rs4759314 and rs12826786 on lncRNA *HOTAIR* are associated with susceptibility to lung cancer in the Chinese Han population [32]. The SNP rs13254990 on lncRNA *PVT1* is associated with the risk of lung adenocarcinoma [33]. However, the correlation between functional SNPs in the identified lncRNAs and susceptibility to silicosis has not been explored in previous studies.

Therefore, a multi-stage study was conducted to explore the relationship between SNPs on lncRNAs and susceptibility to silicosis. First, to identify differentially expressed lncRNAs (DE-lncRNAs), RNA-sequencing (RNA-seq) was conducted based on four pairs of silicosis cases and silica-exposed controls. Second, we systematically selected the functional SNPs in the DE-lncRNAs using several databases and bioinformatics analyses. Third, a two-stage case-control study was designed to evaluate the relationship between functional SNPs and susceptibility to silicosis.

## Materials and methods

### Study design

**Fig. 1:**
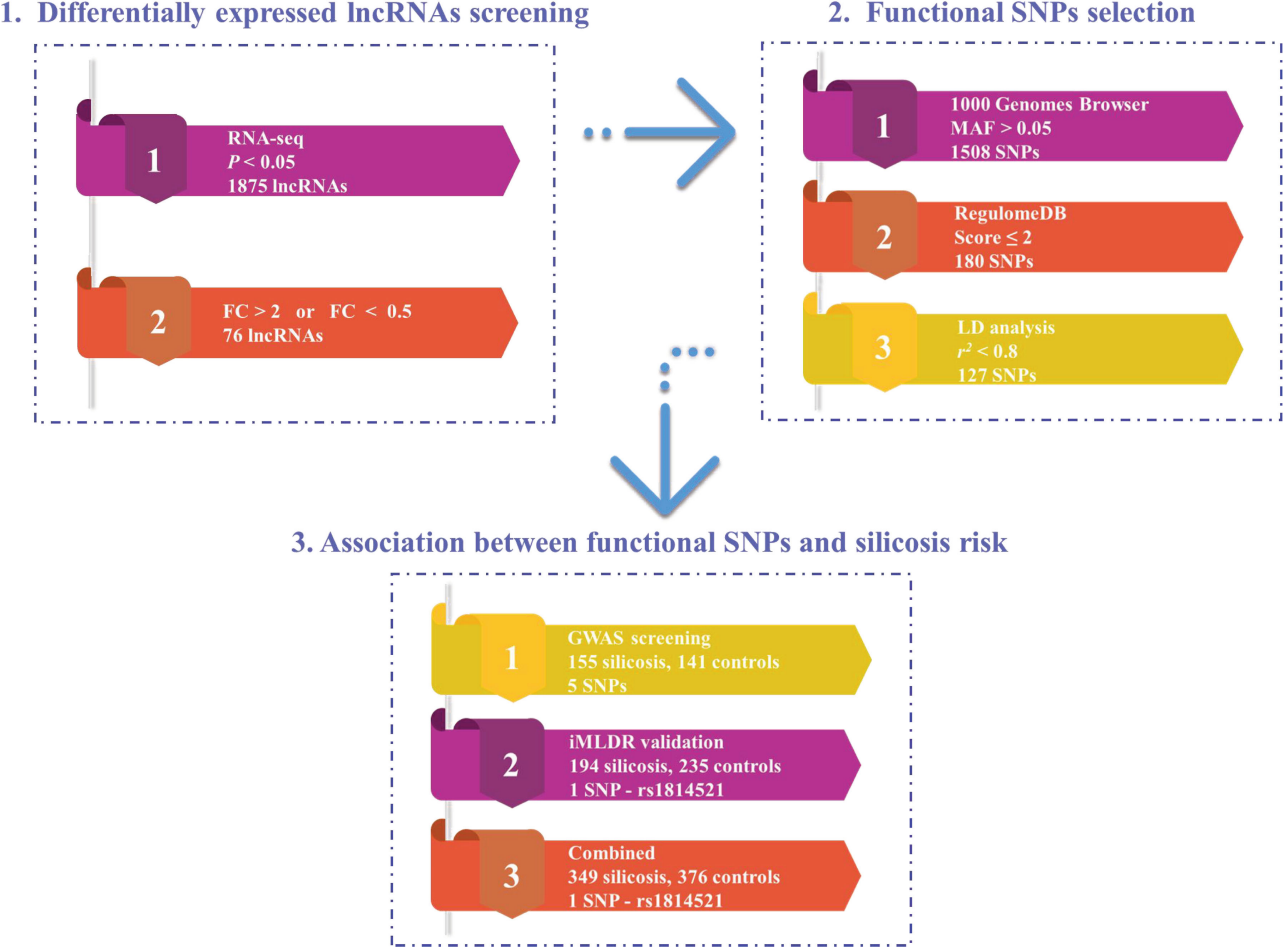
Schematic representation of the multi-stage study design. RNA-seq, RNA sequencing; MAF, minor allele frequency; LD analysis, linkage disequilibrium analysis

A multi-stage study was conducted to explore the correlation between functional SNPs in lncRNAs and susceptibility to silicosis (Fig. 1). First, RNA-seq data on peripheral blood lymphocytes (PBLs) from four silicosis cases and four healthy controls (matched by the number of years the cases were exposed to silica dust) were comprehensively analyzed to obtain the DE-lncRNAs. Second, the SNPs located in DE-lncRNAs were screened using the 1000 Genomes Browser database, and the functions of the SNPs were evaluated using the regulome DB database. Finally, to study the correlation between the functional SNPs in DE-lncRNAs with susceptibility to silicosis, a two-stage case-control study matched by the number of years the cases were exposed to silica dust was conducted (GWAS screening stage: 155 silicosis cases and 141 healthy silica-exposed controls; validation stage: 194 silicosis cases and 235 healthy silica-exposed controls). The study protocol was approved by the Ethics Committee of the Center for Disease Control and Prevention of Wuxi (Approval No. 2020-81), and written informed consent was obtained from all participants.

### Study population

For the identification of DE-lncRNAs, four silicosis cases and four healthy controls were selected in September 2017 from the Wuxi Institute of Occupational Diseases for the identification (Supplementary Table 1).

For the population susceptibility study, in the GWAS screening stage, 155 silicosis cases were recruited from 2012 to 2016 in the Wuxi Institute of Occupational Diseases. One-hundred and forty-one healthy controls were randomly selected from a pool of >2000 occupational silica-dust-exposed individuals who participated in a routine health surveillance at the Wuxi Institute of Occupational Diseases in 2017. The healthy controls were matched with the cases based on the number of years of silica dust exposure.

For validation of the GWAS results using the improved Multiligase Detection Reaction (iMLDR) system, 194 cases of silicosis were recruited in 2017 from Datun Mining Business Group Co. Ltd. of Xuzhou in Jiangsu Province. Moreover, 235 healthy controls were randomly selected from a pool of >1000 occupational silica-dust-exposed individuals who participated in a routine health surveillance in 2017 in Xuzhou.

Each study subject was interviewed by well-trained staff using a structured questionnaire, which included questions on all demographic characteristics relevant to the study.

### RNA-seq screening for DE-lncRNAs

Total RNA was extracted from the PBLs of four silicosis cases and four matched healthy silica-exposed controls using TRIzol reagent (Invitrogen, Carlsbad, CA, USA) and a miRNeasy mini kit (Qiagen, Hilden, Germany), following the manufacturer’s instructions. Then, the RNA samples were sent to Gminix, Biotechnology Co. Ltd. (Shanghai, China) for RNA-seq. The samples were sequenced on an Illumina HiSeq 2500 sequencing platform with an average of 15G reads. DE-lncRNAs between the four silicosis cases and four healthy silica-exposed controls were identified using the following criteria: *P* < 0.05 and fold change (FC) > 2 (cases/controls: >2-fold upregulated or <0.5-fold downregulated).

### Selection of functional SNPs in candidate DE-lncRNAs

First, the 1000 Genomes Project (http://www.1000genomes.org/) was employed to screen the SNPs that were located on the above-identified DE-lncRNAs. Then, the candidate SNPs with a minor allele frequency (MAF) > 0.05 in the Chinese Han population were further screened out.

Subsequently, the RegulomeDB database (http://www.regulomedb.org/index) was employed to evaluate the potential regulatory functions of the candidate SNPs with RegulomeDB scores ranging from 1a to 2c. Furthermore, candidate SNPs, with a linkage disequilibrium (LD) value (*r*^2^ > 0.8), were filtered.

### Genotyping platform of the two-stage case-control study

Genomic DNA was extracted from the peripheral blood using a DNA Extraction Kit (Qiagen, Valencia, CA, USA), following the manufacturer’s instructions. The GWAS was constructed with Illumina Asian Screening Array chip and standard quality control procedures were performed before association analysis and a total of 746,113 SNPs were genotyped in the GWAS in 155 silicosis cases and 141 healthy controls. In the validation stage, the SNPs were genotyped using the Genesky proprietary improved Multiligase Detection Reaction (iMLDR) multiplex SNP genotyping system. The iMLDR genotyping system employs a multiplex PCR-ligase detection reaction method. For each SNP, the alleles are distinguished using different fluorescent labels of allele-specific oligonucleotide probe pairs. Then, different SNPs are further distinguished according to different extended lengths at the 3’end.

### Statistical analyses

Differences in the distribution of demographic characteristics and selected variables between cases and controls were calculated using two-sided χ^2^ tests or Student’s t-tests. The Student’s t-test is used for continuous variables when the data met the assumptions of normality, independence, and homogeneity of variance. The Chi-square test is applied to the statistical inference of categorical variables. Logistic regression analysis was conducted to check the correlation between candidate SNPs and silicosis risk based on odds ratios (ORs) and 95% confidence intervals (CIs), which were adjusted for sex, age, smoking status, and years of exposure to silica dust. Statistical analyses were performed using SPSS version 20.0, STATA version 12.0, or R version 3.6.2 software, where appropriate.

## Results

### Characteristics of the study participants

The characteristics of the 155 silicosis cases and 141 healthy silica-exposed controls included in the screening stage and 194 silicosis cases and 235 healthy silica-exposed controls included in the validation stage are shown in Table 1. In both stages, no significant differences were observed in terms of the number of years of silica dust exposure and sex ratio between cases and controls (*P* > 0.05).

**Table 1:** Characteristics of the subjects enrolled in this study

**Variables**	**Screening (GWAS)**			**Validation (iMLDR)**		
	
	**Case**	**Control**	***P***	**Case**	**Control**	***P***
	**(N = 155)**	**(N = 141)**		**(N = 194)**	**(N = 235)**	
Age, years (mean ± SD)	67.53 ± 8.24	60.25 ± 6.31	<0.001	68.73 ± 9.01	62.71 ± 11.74	<0.001
Exposure years (mean ± SD)	24.80 ± 7.00	23.72 ± 5.55	0.146	27.13 ± 8.16	23.58 ± 8.50	0.055
Sex, N (100%)			0.051			0.151
Male	138(89.03)	114(80.85)		189(97.42)	222(94.47)	
Female	17(10.97)	27(19.15)		5(2.58)	13(5.53)	
Smoking status, N (100%)			0.019			0.005
Ever	98(63.23)	70(49.65)		103(53.09)	92(39.15)	
Never	57(36.77)	71(50.35)		91(46.91)	143(60.85)	
Stage, N (100%)						
I	94(60.65)			154(79.38)		
II	51(32.90)			28(14.43)		
III	10(6.45)			12(6.19)		

### Identification of DE-lncRNAs through RNA-seq

**Fig. 2:**
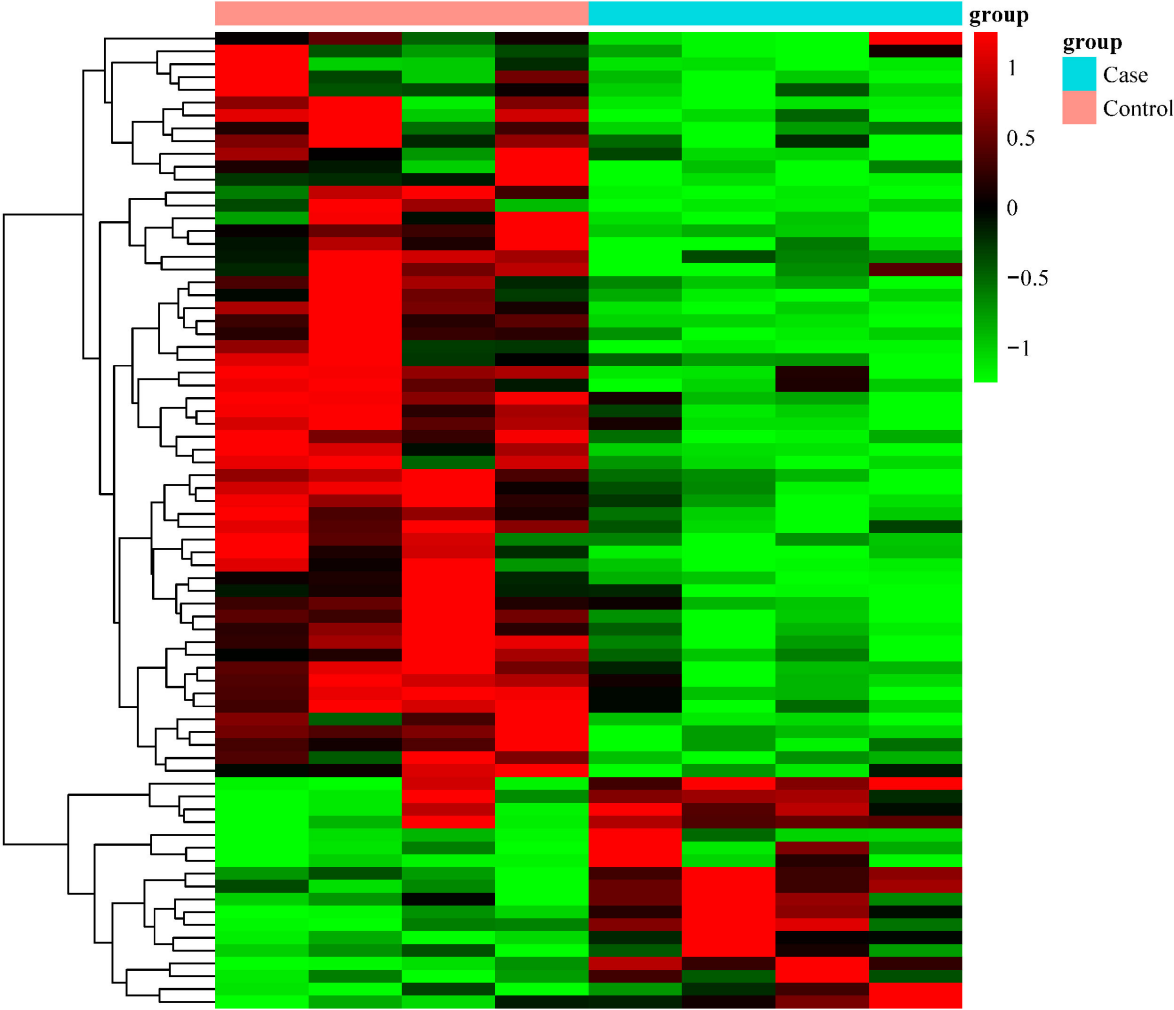
Hierarchical clustering analysis of the 18 upregulated and 58 downregulated long non-coding RNAs. The fold change and *P*-value were calculated using the *limma* package in R.

A total of 1,875 DE-lncRNAs were identified between four silicosis cases and four healthy silica-exposed controls (*P* < 0.05) by RNA-seq analysis in the PBLs. Among the 1,875 DE-lncRNAs, 76 DE-lncRNAs with FC > 2 or < 0.5 were selected. Among the 76 DE-lncRNAs, 58 lncRNAs were downregulated, and 18 lncRNAs were upregulated in the silicosis cases compared with the healthy controls (Fig. 2).

### Selection of functional SNPs in candidate DE-lncRNAs

Based on the above 76 DE-lncRNAs, the 1000 Genome Project was used to search for all SNPs located on those 76 DE-lncRNAs. The results suggested that 24,653 SNPs were located in the 76 DE-lncRNA regions. Subsequently, 1,508 SNPs with a MAF value > 0.05 were obtained for the Han Chinese population.

The RegulomeDB database demonstrates a scoring system ranging from 1 to 6. A lower RegulomeDB score indicates a higher likelihood that the variant is located in a particular functional area. Thus, we further screened the 1,508 SNPs using the RegulomeDB database and selected SNPs with RegulomeDB scores lower than 3, finally obtaining a total of 180 SNPs. After performing linkage disequilibrium (LD) analysis (*r*^2^ < 0.8), a total of 127 SNPs were finally obtained.

### Association between functional SNPs and silicosis risk

To further explore the relationship between the 127 SNPs and the risk of developing silicosis, we evaluated the effect of these SNPs on silicosis susceptibility from the results of GWAS. The results showed that among these 127 SNPs, five SNPs were significantly correlated with the risk of silicosis (*P* < 0.05) (Table 2).

**Table 2:** Characteristics of the five SNPs in this study

**Number**	**SNPs**	**lncRNA**	**Chr**	**Alleles**	**Cases** **(N = 155)**	**Controls** **(N = 141)**	**MAF** **(Cases)**	**MAF** **(Controls)**	**OR** **(95%CI)^a^**	***P***
1	rs226235	*LASP1*	chr17:37044029	T>C	89/58/8	61/67/13	0.239	0.330	0.54(0.34–0.87)	0.011
2	rs12952054	*LASP1*	chr17:37054253	A>G	118/36/1	87/53/1	0.123	0.195	0.39(0.21–0.74)	0.004
3	rs1814521	*ADGRG3*	chr16:57716865	G>A	54/75/25	38/66/36	0.406	0.493	0.64(0.42–0.97)	0.037
4	rs67331468	*PYURF*	chr4:89444271	G>A	41/75/39	42/68/31	0.494	0.461	1.85(1.11–3.06)	0.017
5	rs2029345	*LASP1*	chr17:37050575	A>G	82/65/8	64/62/15	0.261	0.326	0.58(0.36–0.93)	0.025

^a^Logistic regression analysis adjusted for age, gender, years of silica dust exposure and smoking status in the additive model (OR; CI).

**Fig. 3:**
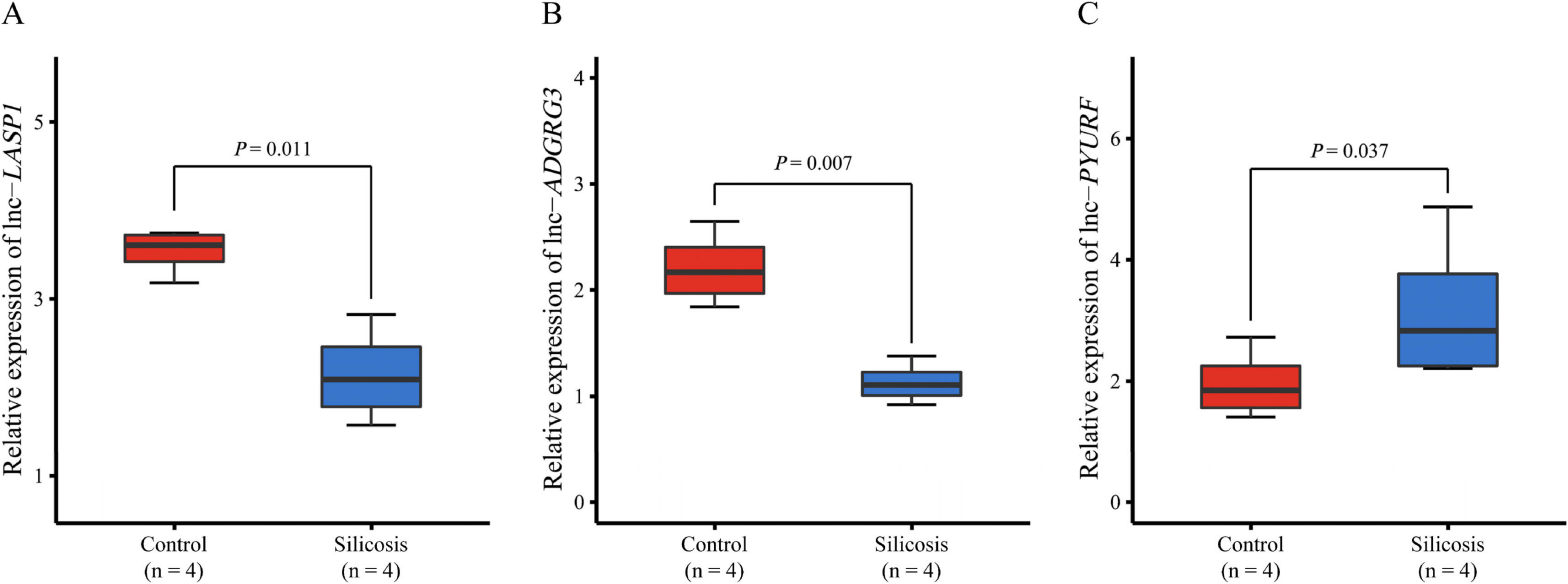
Differential expression of **(A)** *lnc-LASP1*, **(B)** *lnc-ADGRG3*, and **(C)** *lnc-PYURF* between silicosis cases and healthy controls. The *P*-value was calculated using the *limma* package in R.

Among the above five SNPs, three SNPs (rs226235, rs12952054, and rs2029345) were located in the genomic region of lncRNA *LASP1*, while rs1814521 and rs67331468 were located in the genomic regions of lncRNA *ADGRG3* and *PYURF*, respectively. Differences in the expression patterns of the above three lncRNAs are shown in Fig. 3.

The iMLDR multiplex SNP genotyping system was employed to further validate the correlation between the above-mentioned five candidate SNPs and the risk of silicosis. The result showed that the variant A allele of rs1814521 was nominally associated with a reduced risk of silicosis compared with the wild G allele (additive model: OR = 0.77, 95% CI = 0.59–1.01, *P* = 0.060) (Table 3).

**Table 3:** Specific information of rs1814521 in different stages

**Stage**	**Genotypes**	**Cases, N (100%)**	**Controls, N (100%)**	**Adjusted OR^b^ (95%CI)**	***P***
Screening	GG	54(35.07)	38(27.14)	1(ref)	
	GA	75(48.7)	66(47.14)	0.79(0.46–1.35)	0.393
	AA	25(16.23)	36(25.72)	0.51(0.26–0.99)	0.047
	Dominant model			0.69(0.42–1.15)	0.154
	Recessive model			0.59(0.33–1.05)	0.071
	Additive model			0.64(0.42–0.97)	0.037

Validation	GG	82(42.27)	82(34.89)	1(ref)	
	GA	84(43.30)	105(44.68)	0.83(0.54–1.27)	0.381
	AA	28(14.43)	48(20.43)	0.57(0.33–1.01)	0.056
	Dominant model			0.75(0.50–1.11)	0.149
	Recessive model			0.64(0.38–1.07)	0.088
	Additive model			0.77(0.59–1.01)	0.060

Combined	GG	136(39.08)	120(32.00)	1(ref)	
	GA	159(45.69)	171(45.60)	0.83(0.60–1.16)	0.280
	AA	53(15.23)	84(22.40)	0.56(0.37–0.86)	0.008
	Dominant model			0.74(0.55–1.01)	0.061
	Recessive model			0.62(0.42–0.92)	0.016
	Additive model			0.76(0.62–0.94)	0.011

^b^Logistic regression analysis adjusted for age, gender, years of silica dust exposure and smoking status.

We further integrated the results of GWAS screening and iMLDR validation and found that the variant A allele of rs1814521 was associated with a reduced risk of silicosis compared with the G allele (additive model: OR = 0.76, 95% CI = 0.62–0.94, *P* = 0.011) (Table 3).

## Discussion

In the present study, we first identified 76 DE-lncRNAs between silicosis cases and healthy controls through RNA-seq screening. Then, 127 functional SNPs were identified from the 76 DE-lncRNAs through multiple public databases. After evaluating the correlation between the above 127 functional SNPs and the susceptibility to silicosis through GWAS, five SNPs were screened. Furthermore, the results of iMLDR validation indicated that among the five SNPs, rs1814521 located in the lncRNA *ADGRG3* was associated with silicosis risk. The combined results of GWAS and iMLDR genotyping indicated that the variant A allele of rs1814521 was associated with a lower risk of silicosis than the wild G allele. Taken together, we revealed that the variant A allele of rs1814521 located in the lncRNA *ADGRG3* was associated with a decreased risk of silicosis.

LncRNAs, a subgroup of ncRNAs, were initially considered as ‘transcriptional noise’ devoid of biological functions. However, with advances in high-throughput sequencing technologies, the important roles of lncRNAs in genetic and epigenetic regulation as well as transcriptional and post-transcriptional regulation have been increasingly recognized [34]. Moreover, several studies have explored the role of lncRNAs as biomarkers in various fibrotic diseases. For example, Zhang et al. found that *lnc-Hser* could enhance the epithelial-mesenchymal transition (EMT) process and increase the rate of apoptosis of hepatocytes, thus indicating that *lnc-Hser* is a promising biomarker of hepatic fibrosis [35]. Moreover, lncRNA H19 knockdown could reduce the progression of pulmonary fibrosis via the lncRNA H19-miR-140-TGF-β/Smad3 regulatory network, suggesting that lncRNA H19 may serve as an early diagnostic and prognostic biomarker for pulmonary fibrosis [36]. In the present study, lncRNA *ADGRG3* was found to be significantly downregulated in silicosis cases compared to the healthy controls, indicating that lncRNA *ADGRG3* may play a role in the development of silicosis.

*ADGRG3*, also known as *GPR97*, is a member of the adhesion G protein-coupled receptor (*ADGRG*) family. The *ADGRG* family is involved in a variety of biological functions [37, 38]. For example, Lin et al. demonstrated that the *ADGRG* family could regulate immunization and inflammation [39]. Therefore, the decreased expression of *ADGRG3* may reduce the development of fibrosis by inhibiting inflammation. Meanwhile, *ADGRG3* was found to be upregulated during systemic inflammation [40], thus supporting our hypothesis. Moreover, the number of macrophages that invaded the liver and kidney was increased after *GPR97* knockout in high-fat diet-induced obese mice [41]. This study revealed that although *GPR97* might enhance inflammation in the liver and kidney, it does not induce metabolic disorders under conditions of high-fat diet-induced obesity. Studies have also shown that *ADGRG3* could regulate the activity of several signaling pathways. For example, Fang et al. revealed that the downregulation of *GPR97* was associated with reduced activity of Sema3A [42], and Jeon et al. showed that upregulated Sema3A expression could promote the development of stromal fibrosis [43]. Additionally, *ADGRG3* has been shown to inhibit NF-κB signaling [44], whereas activated NF-κB signaling further promotes alveolar epithelial cell senescence, which ultimately leads to pulmonary fibrosis [45]. Therefore, these studies indicate that *ADGRG3* may reduce the progression of fibrosis by inhibiting the activity of Sema3A and NF-κB signaling.

There are several advantages to this study. First, we systematically screened the DE-lncRNAs related to silicosis and further explored the correlation between these DE-lncRNA- related SNPs and susceptibility to silicosis, the two-stage screening process greatly increases the reliability of our results. To the best of our knowledge, the correlation between these SNPs and the risk of silicosis has not been explored in previous studies. Second, the identified SNPs were genotyped by a two-stage study (GWAS screening: 155 cases vs. 141 controls; iMLDR validation: 194 cases vs. 235 controls). The results of combined GWAS and iMLDR genotyping revealed a significant correlation between the SNPs and silicosis risk (Combination: 349 cases vs. 376 controls). However, our study still has some limitations. First, the cases and controls included in our study were matched mainly in terms of the number of years of silica dust exposure. Other factors, including age and smoking status, were not comparable between the cases and controls. Although the ORs and 95% CIs were adjusted for age, sex, and smoking status in the logistic regression analysis, one cannot rule out the possibility that these factors may affect our results. Therefore, controls should be strictly matched with cases in terms of the above-mentioned factors in future studies. Second, to include more potential functional SNPs for validation, we appropriately relaxed the inclusion criteria during the GWAS stage and did not correct the *P*-value when performing multiple comparisons. Although the iMLDR validation stage may enhance the statistical significance of our study, further studies with corrected *P*-values by multiple comparisons should be conducted. Third, we performed RNA-seq of PBLs, which account for only 40% of peripheral blood. Therefore, PBLs are not the complete representative of peripheral blood, and hence the expression of *ADGRG3* in whole blood needs to be evaluated in subsequent studies.

## Conclusion

The functional SNP rs1814521 in lncRNA *ADGRG3* could affect the risk of silicosis and *ADGRG3* was lowly expressed in silicosis cases. Future studies should aim to elucidate the mechanisms underlying the effects of lncRNA *ADGRG3* and rs1814521 in silicosis development.

## Supplementary information

**Additional file 1: Supplementary Table 1** The basic characteristics of the 8 subjects whose PBL-RNA was sequenced.
