# Supplementary material for: The single nucleotide polymorphism rs1814521 in long non-coding RNA ADGRG3 associates with the susceptibility to silicosis: a multi-stage study
Source: Environ Health Prev Med. 2022 Feb 19;27:5. doi: 10.1265/ehpm.21-00338 (PMC9093617; doi:10.1265/ehpm.21-00338)
Supplement: Supplementary file 1 — Additional file 1: Supplementary Table 1 The basic characteristics of the 8 subjects whose PBL-RNA was sequenced. [file ehpm-27-005-s001.docx]

**Supplementary Table 1** The basic characteristics of the 8 subjects whose PBL-RNA was sequenced

| Variables | RNA-seq screening | | |
| --- | --- | --- | --- |
|  | controls  (n = 4) | silicosis  (n = 4) | *P* |
| Age (mean ± SD) | 61.75 ± 7.59 | 64.75 ± 7.81 | 0.60 |
| Exposure years (mean ± SD) | 20.50 ± 6.56 | 24.00 ± 6.06 | 0.46 |
| Sex, n (100%) |  |  | -- |
| Male | 4 (100) | 4 (100) |  |
| Female | 0 | 0 |  |
| Smoking status, n (100%) |  |  | 0.43 |
| Never | 2 (50.00) | 0 |  |
| Ever | 2 (50.00) | 4 (100) |  |
| Stage, n (100%) |  |  | -- |
| I |  | 4 (100) |  |
| II |  | 0 |  |
| III |  | 0 |  |
